# Prevalence and risk factors for vitamin D insufficiency in 6-12-month-old infants: a cross-sectional study in Southern Thailand

**DOI:** 10.1186/s12887-022-03797-y

**Published:** 2022-12-21

**Authors:** Staporn Kasemsripitak, Somchit Jaruratanasirikul, Sasivara Boonrusmee, Tansit Saengkaew, Hutcha Sriplung

**Affiliations:** 1 Department of Pediatrics, Faculty of Medicine, Prince of Songkla University, Hat Yai, Thailand; 2 Division of Pediatric Endocrinology, Department of Pediatrics, Faculty of Medicine, Prince of Songkla University, Hat Yai, Songkhla, 90110 Thailand; 3 Division of Ambulatory Pediatrics, Department of Pediatrics, Faculty of Medicine, Prince of Songkla University, Hat Yai, Thailand; 4 Epidemiology Unit, Faculty of Medicine, Prince of Songkla University, Hat Yai, Thailand

**Keywords:** Infancy, Sunlight exposure, Vitamin D insufficiency, Vitamin D status

## Abstract

**Background:**

Vitamin D is an essential micronutrient for bone mineralization and bone growth in children. There have been few studies to date of vitamin D status in infants aged 6–12 months in Southeast Asian countries.

**Aim:**

To examine the prevalence of vitamin D insufficiency (VDI, including vitamin D deficiency) in healthy infants and the risk factors for VDI in southern Thailand.

**Methods:**

A cross-sectional study was carried out in 120 healthy infants aged 6–12 months and their mothers. Blood samples were obtained for measurement of serum calcium, phosphate, alkaline phosphatase, albumin, parathyroid hormone and 25-hydroxyvitamin D (25OHD) levels. The mothers were interviewed for their infants feeding intake, sunlight exposure, type of dress, and sunscreen use. Chi-square and *t-test* were used to compare between groups for categorical and continuous variables, respectively. Pearson correlation was used to identify the relationship between serum levels of 25OHD of the infants and other biochemical variables of the infants and serum levels of maternal 25OHD. Logistic regression analysis was used to identify the factors associated with vitamin D status.

**Results:**

The prevalence of VDI in our study infants was high at 28.3%, all of whom were mainly breastfed infants. Subgroup analysis of the mainly breastfed infants found that the risk factors for VDI were maternal VDI and a short duration of sunlight exposure. The serum 25OHD levels of the mainly breastfed infants were significantly positively correlated with the maternal serum 25OHD levels (*r* = 0.49, *p-value* < 0.01) and with sunlight exposure duration (*r* = 0.40, *p-value* < 0.01).

**Conclusions:**

The prevalence of VDI was high in 6–12-month-old infants, particularly those who were mainly breastfed from VDI mothers, and who had short durations of sunlight exposure. As breast milk contains adequate amounts of most important vitamins and growth factors, breastfeeding is still encouraged for infants with 400 IU vitamin D daily supplementation to prevent VDI.

## Introduction

Vitamin D is an essential micronutrient in children for bone mineralization and bone growth by regulation of calcium and phosphate metabolism [1]. In infants, breast milk is highly nutritive containing adequate amounts of most important vitamins and immunological and growth factors [2]. The World Health Organization and the Academy of Nutrition and Dietetics recommend exclusive breastfeeding for the first six months of life and breastfeeding with complementary food from six months until at least 12 months of age as the ideal feeding pattern for infants [3, 4]. In Thailand, The Royal College of Pediatricians of Thailand recommends exclusive breastfeeding for infants from birth to at least 6 months of age and that breastfeeding should be continued along with complementary food to 2 years of age [5]. However, human breast milk has been found to be low in certain nutrients such as vitamin D, iron, and vitamin K [6, 7].

The main source of vitamin D is cutaneous vitamin D synthesis from sunlight exposure. The peak time for cutaneous vitamin D synthesis is during 10.00–15.00 h. Despite an abundance of sunshine for most of the year in Southeast Asian countries including Thailand, various studies by South East Asian Nutrition Surveys (SEANUTS) have found more than 30% of children and adolescents with vitamin D insufficiency [8, 9]. There have been few studies to date of vitamin D status in infants aged 6–12 months in Southeast Asia.

This study was performed to examine vitamin D status in healthy infants aged 6–12 months attending the Well Baby Clinic at Songklanagarind Hospital, the main tertiary care center and medical school hospital in Southern Thailand. It is located at 780 km (485 miles north of the equator with an average of 6 h of sunshine per day, only 2 seasons (summer and rainy) with minimal seasonal variation, and an average year-round temperature of 24–34 degrees Celsius [10]. The main purpose of the study was to examine the prevalence of vitamin D insufficiency in healthy Thai infants using serum 25OHD levels. The secondary outcomes were to determine the risk factors related to vitamin D insufficiency, including feeding and dressing patterns, timing and duration of sunlight exposure, and maternal serum 25OHD levels.

## Subjects and methods

### Sample size calculation and ethics considerations

The sample size calculation was based on the reported prevalence of vitamin D insufficiency in our region in the SEANUTS studies in children aged 3–10 years [8, 9] of 30% with a study power of 80%. The required sample size was 95 infants.

The protocol for this study was approved by the Institutional Review Board and the Ethics Committee of Songklanagarind Hospital, Prince of Songkla University (REC.63–358-1–1). Written informed consent was obtained from the parents of each study subject.

### Participants

From December 2020 to November 2021, 120 pairs of healthy infants aged 6–12 months and their mothers who attended the Well Child Clinic of Songklanagarind Hospital were enrolled. Infants with a history of preterm birth or term birth with an underlying disease such as congenital heart disease, respiratory, gastrointestinal, liver or renal diseases, etc. were excluded. The demographic data of the mother and the characteristics of the infant were collected. The weight of each infant was measured without clothing using a digital weighing scale (Seca, Model 882 GmbH, Hamburg, Germany) to the nearest 0.1 kg. The body length was measured with the infant in a lying position with an infantometer to the nearest 0.1 cm. Head circumference was measured in the occipito-frontal line using a non-elastic plastic tape to the nearest 0.1 cm. All measurements of weight, length and head circumference were transformed to a z-score following the World Health Organization (WHO) database [11].

### Feeding, sunlight exposure, dress and use of sunscreen

The mothers were interviewed for their infants feeding habits including breast feeding and formula feeding (frequency and amount of milk intake), complementary food, sunlight exposure (timing and duration of exposure per day and number of days exposure per week), use of sunscreen and the area(s) the sunscreen was applied, and type of dress of the infant. The type of milk intake was classified as mainly breastfed or mainly formula-fed depending on which was given more than 50% of total daily milk intake. The duration of daily sunlight exposure was assessed using a structured questionnaire about the time of the day that the infants were brought outdoors and the overall daily duration in minutes of sunlight exposure and the days per week of sunlight exposure. The duration of sunlight exposure time was calculated in minutes per week.

### Biochemical measurements

A 3-mL blood sample was obtained from both the mother and the infant for measurement of serum calcium, phosphate, albumin, alkaline phosphatase (ALP), parathyroid hormone (PTH) and 25-hydroxyvitamin D (25OHD) levels. Serum 25OHD levels were measured using a chemiluminescence immunoassay (Liaison®; DiaSorin, Stillwater, MN). The intra-assay and inter-assay coefficients of variation for the serum 25OHD levels were 3.9 and 5.5%, respectively. The PTH levels were measured by electrochemiluminescent assay. Serum calcium, phosphorus, albumin, and alkaline phosphatase were measured by calorimetric assay (Cobas, Roche Diagnostics, Indianapolis, Indiana, USA).

Following the Global Consensus Recommendations on Prevention and Management of Nutritional Rickets, we defined vitamin D deficiency (VDD), vitamin D insufficiency (VDI) and vitamin D sufficiency (VDS) as serum 25OHD concentrations of < 12, 12–20 and > 20 ng/mL, respectively [12].

### Statistical analysis

All statistical analyses were performed using the R program (R Foundation, Austria, available from http://www.r-project.org/foundation/main.html). The demographic data were expressed in numbers and percentages (categorical variables). The continuous variables were calculated as means ± standard deviations.

The numbers and proportions of children and mothers with low and adequate serum 25OHD concentrations were compared using the chi-square test and biochemical levels were compared by *t-test*. Pearson correlation was used to identify the relationship between serum levels of 25OHD of the infants and other biochemical variables of the infants and serum levels of maternal 25OHD. Logistic regression analysis was used to identify the factors associated with vitamin D status. Significance was set at *p-value* < 0.05.

## Results

### Participant characteristics

The average age of the infants at the time of the study was 7.2 ± 1.7 months with average weight, length and head circumference of 7.9 ± 1.1 kg, 68 ± 3 cm and 43.3 ± 1.6 cm, respectively. The characteristics of the mothers and the infants with the z-scores of weight, length and head circumference are shown in Table 1. None of the infants or mothers were on medications or vitamin supplementation.

**Table 1:** Characteristics of the 120 study participants, data are shown in mean ± SD or n (%) as appropriate

**Characteristic**	**Mean** ± **SD**	**Range**
**Infants**		
Male, n (%)	70 (58)	-
Age (months)	7.2 ± 1.7	6—12
Weight (kg)	7.9 ± 1.1	5.3—10.6
Length (cm)	68 ± 3	60—77
Head circumference (cm)	43.3 ± 1.6	39—46
Weight z-score	0.45 ± 1.30	-2.28—3.71
Length z-score	0.56 ± 0.99	-1.83—3.40
Head circumference z-score	-0.07 ± 0.93	-2.46—2.92
Main milk feeding, n (%)		
Breast feeding	61 (51)	-
Formula feeding	59 (49)	-
Main caregiver, n (%)		
Parents (mother ± father)	95 (79)	-
Grandparents	25 (21)	-
Dress, n (%)		
Short-sleeved shirts and short pants	100 (83)	-
Long-sleeved shirts and long pants	20 (17)	-
Days of sunlight exposure per week, n (%)		
6–7 days	65 (54)	-
4–5 days	25 (21)	-
2–3 days	16 (13)	-
1 day	14 (12)	-
Duration of sun exposure per day (minutes)	15 ± 9	2—30
Duration of sun exposure per week (minutes)	85 ± 46	5—210
Time of exposure, n (%)		
Before 10.00 h	84 (70)	-
During 10.00–15.00 h	4 (3)	-
After 15.00 h	32 (27)	-
**Mothers**		
Age (years)	33 ± 5	22—42
Weight (kg)	58 ± 15	39–90
Height (cm)	159 ± 6	147—172
Level of education, n (%)		
Bachelor	93 (77)	-
High school	27 (23)	-
Family income (Baht per month)*	40,000 ± 25,000	15,000—180,000

### Main feeding intake, dress, sunlight exposure and use of sunscreen

The main milk intake of the infants was from breast feeding in 61 infants (51%) and formula feeding in 59 (49%). The average volume of milk intake in the mainly formula-fed infants was 888 ± 181 mL (range 600–1440). For type of infant dress, 100 (83%) were normally dressed in short-sleeved shirts and short pants. The preferred time the caregivers brought their infants for outdoor activities was in the morning 07.30–08.30 h (n = 84, 70%) or in the late afternoon 16.30–17.30 h (n = 32, 27%) with only 4 infants (3%) who were brought outdoors during 10.00–15.00 h. The average duration of sunlight exposure of the infants was 15 ± 9 min per day, on an average of 5.5 days per week. The average duration of sunlight exposure per week of the infants was thus 85 ± 46 min. None of the infants had sunscreen applied during their outdoor time (Table 2).

**Table 2:** Comparison of participants’ characteristics and biochemical studies between vitamin D insufficiency (VDI) and vitamin D sufficiency (VDS), data are shown in mean ± SD or n (%) as appropriate

	**Total (*N* = 120)**	**VDI (*N* = 34)**	**VDS (*N* = 86)**	***p*-value**
**Infants**				
25OHD (ng/mL)	25.6 ± 9.8	13.4 ± 4.1	30.4 ± 6.6	< 0.01
PTH (pg/mL)	26.3 ± 13.2	31.2 ± 18.6	24.5 ± 9.9	0.06
Calcium (mg/dL)	10.4 ± 0.3	10.3 ± 0.3	10.4 ± 0.3	0.48
Phosphate (mg/dL)	5.6 ± 0.5	5.3 ± 0.4	5.7 ± 0.5	< 0.01
Albumin (g/dL)	4.4 ± 0.2	4.5 ± 0.2	4.4 ± 0.2	0.54
Alkaline phosphatase (IU/L)	279 ± 71	270 ± 62	283 ± 74	0.35
**Mothers**				
25OHD (ng/mL)	23.1 ± 6.0	20.2 ± 4.6	24.2 ± 6.2	< 0.01
Parathyroid hormone (pg/mL)	38.8 ± 16.8	38.1 ± 17.8	39.1 ± 16.5	0.79
Calcium (mg/dL)	9.3 ± 0.4	9.4 ± 0.4	9.3 ± 0.4	0.12
Phosphate (mg/dL)	3.6 ± 0.5	3.7 ± 0.5	3.5 ± 0.5	0.07
Albumin (g/dL)	4.5 ± 0.2	4.6 ± 0.2	4.5 ± 0.2	0.54
Alkaline phosphatase (IU/L)	89 ± 29	96 ± 25	86 ± 30	0.06
**Characteristic**				
Gender male**,** n (%)	70 (58.3)	21 (61.8)	49 (57.0)	0.63
Weight (kg)	7.9 ± 1.1	7.7 ± 1.1	7.9 ± 1.2	0.29
Length (cm)	68.2 ± 3.3	67.5 ± 3.2	68.5 ± 3.3	0.12
Head circumference (cm)	43.3 ± 1.6	43.0 ± 1.6	43.4 ± 1.5	0.32
Weight z-score	0.45 ± 1.30	0.32 ± 1.23	0.50 ± 1.28	0.50
Length z-score	0.56 ± 0.99	0.36 ± 0.97	0.64 ± 0.99	0.16
Head circumference z-score	-0.07 ± 0.93	-0.20 ± 1.04	-0.02 ± 0.88	0.38
Mainly breastfed, n (%)	61 (52)	34 (100)	27 (31)	< 0.01
Duration of sunlight exposure per day (minutes)	15 ± 9	11 ± 7	16 ± 10	< 0.01
Duration of sunlight exposure per week (minutes)	85 ± 65	54 ± 22	100 ± 25	< 0.01
Time of exposure, n (%)				0.30
Before 10.00 h	84 (70)	21 (62)	63 (73)	
During 10.00–15.00 h	4 (3)	2 (6)	2 (2)	
After 15.00 h	32 (27)	11 (32)	21 (25)	
Dress type, short-sleeved shirts and short pants, n (%)	100 (83)	29 (85)	71 (83)	0.79
Caregivers, parents, n (%)	95 (79)	29 (85)	66 (77)	0.45
Family income (Baht/month)	40,000 ± 24,000	48,000 ± 31,000	37,000 ± 21,000	0.08
Maternal education, bachelor, n (%)	93 (78)	30 (88)	63 (73)	0.18
Maternal VDI, n (%)	41 (34)	18 (53)	23 (27)	< 0.01

### Biochemical measurements and vitamin D status

The average serum 25OHD level of the infants was 25.6 ± 9.8 ng/mL (range 5.6–50.1) and that of the mothers was 23.1 ± 6.0 ng/mL (range 9.4–40.9). VDD, VDI and VDS were classified in 11 (9%), 23 (19%) and 86 (72%) infants and in 1 (1%), 40 (33%) and 79 (66%) mothers, respectively. The average PTH, calcium, phosphate, albumin, and ALP levels in the infants and the mothers were all within normal ranges.

The infants were divided into 2 groups according to vitamin D status, a VDI group (25OHD < 20 ng/mL, which included VDD infants) (n = 34) and a VDS group (n = 86). The average phosphate level was significantly lower in the VDI group than in the VDS group (5.3 ± 0.4 vs 5.7 ± 0.5 mg/dL, *p-value* < 0.01). The average 25OHD level in the mainly formula-fed infants was 31.9 ± 6.6 ng/mL (range 21.4–50.1). The average vitamin D level of the mothers in the VDI group was significantly lower than in the VDS group (20.2 ± 4.6 vs 24.2 ± 6.2 ng/mL, *p-value* < 0.01). The significant differences between the VDI infants and the VDS infants were breastfeeding (all VDI infants were mainly breastfed), maternal vitamin D level, and duration of sunlight exposure per day and per week (Table 2).

### Vitamin D status in mainly breastfed infants

**Fig.1:**
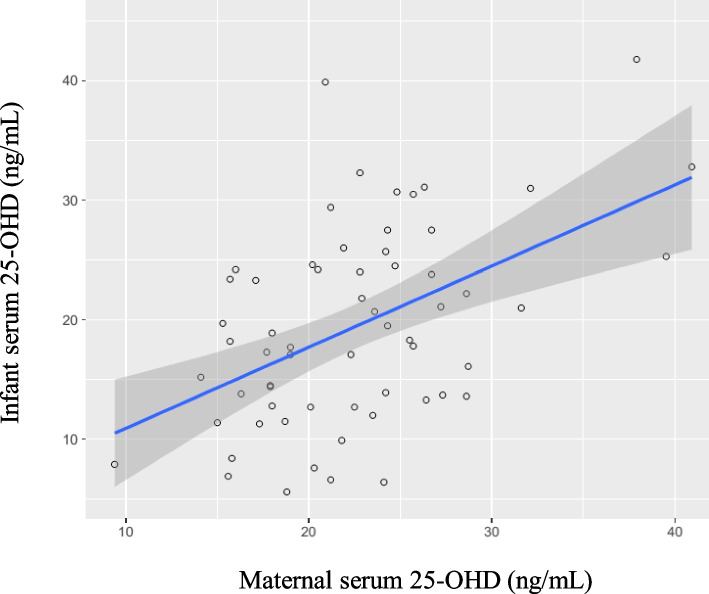
Correlations between maternal serum 25OHD levels and infant 25OHD levels (*r* = 0.49, *p*-value < 0.01)

**Fig. 2:**
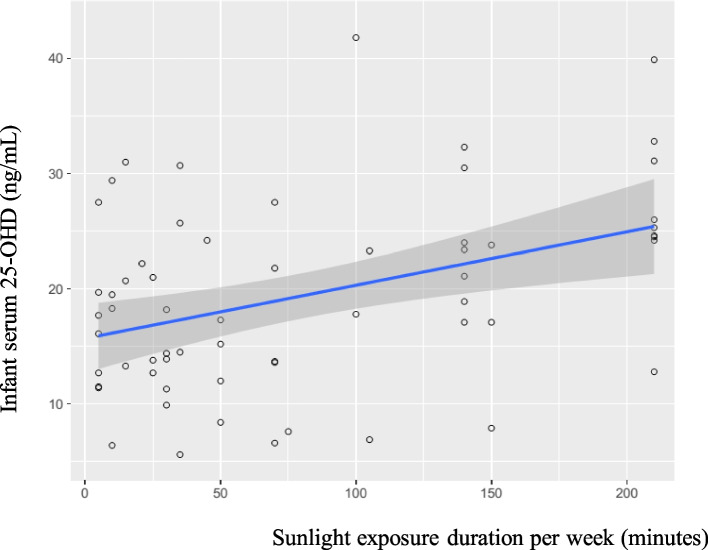
Correlations between infant sunlight exposure duration per week and 25OHD levels in mainly breastfed infants (*r* = 0.40, *p*-value < 0.01)

All the VDI infants were in the mainly breastfed group, but not all breastfed infants had VDI. We further performed subgroup analysis of the mainly breastfed infants to determine the factors associated with VDI (Table 3). Of the 61 mainly breastfed infants, 34 (56%) had VDI and 27 (44%) had VDS. The significantly different variables between the VDI and VDS infants were the durations of sunlight exposure per day and per week, maternal serum 25OHD levels and the percentage of maternal VDI. The serum 25OHD levels of the infants were significantly correlated with the serum levels of the mothers (*r* = 0.49, *p-value* < 0.01) (Fig. 1). The median durations of sunlight exposure per day and per week were significantly lower in the VDI group than in the VDS group (11 ± 7 vs 18 ± 10 min per day and 54 ± 22 vs 114 ± 48 min per week, respectively, *p-values* < 0.01). There was a positive relationship between sunlight exposure duration and 25OHD levels (*r* = 0.40, *p-value* < 0.01) (Fig. 2). By multivariate analysis, the factors significantly associated with VDI were duration of sunlight exposure per week and maternal VDI. (Table 4).

**Table 3:** Comparison of participants’ characteristics and biochemical studies in mainly breastfed infants between vitamin D insufficiency (VDI) and vitamin D sufficiency (VDS), data are shown in mean ± SD or n (%) as appropriate

	**VDI (*N* = 34)**	**VDS (*N* = 27)**	***p* value**
**Infants**			
25OHD (ng/mL)	13.7 ± 5.8	25.3 ± 7.3	< 0.01
Parathyroid hormone (pg/mL)	31.2 ± 20.1	25.0 ± 10.6	0.09
Calcium (mg/dL)	10.3 ± 0.34	10.4 ± 0. 3	0.45
Phosphate (mg/dL)	5.3 ± 0.4	5.4 ± 0.3	0.49
Albumin (g/dL)	4.5 ± 0.2	4.5 ± 0.2	0.40
Alkaline phosphatase (IU/L)	262 ± 89	250 ± 136	0.84
**Mothers**			
25OHD (ng/mL)	19.0 ± 6.8	24.3 ± 6.0	< 0.01
Parathyroid hormone (pg/mL)	38.1 ± 17.8	37.0 ± 17.1	0.89
Calcium (mg/dL)	9.4 ± 0.4	9.4 ± 0.3	0.77
Phosphate (mg/dL)	3.6 ± 0.5	3.8 ± 0.7	0.40
Albumin (g/dL)	4.5 ± 0.2	4.4 ± 0.2	0.40
Alkaline phosphatase (IU/L)	94 ± 31	100 ± 34	0.31
**Characteristic**			
Gender, male**,** n (%)	21 (62)	20 (74)	0.41
Weight (kg)	7.7 ± 1.1	7.9 ± 1.2	0.50
Length (cm)	67.5 ± 3.2	68.8 ± 3.3	0.10
Head circumference (cm)	43.0 ± 1.6	43.1 ± 1.5	0.83
Weight z-score	0.32 ± 1.23	0.36 ± 1.3	0.91
Length z-score	0.36 ± 0.97	0.61 ± 0.91	0.31
Head circumference z-score	-0.20 ± 1.04	-0.33 ± 0.9	0.61
Duration of sunlight exposure per day (minutes)	11 ± 7	18 ± 10	< 0.01
Duration of sunlight exposure per weeks (minutes)	54 ± 22	114 ± 48	< 0.01
Time of exposure, n (%)			0.60
Before 10.00 h	21 (62)	20 (74)	
During 10.00–15.00 h	2 (6)	1 (4)	
After 15.00 h	11 (32)	6 (22)	
Dress type, short-sleeved shirts and short pants, n (%)	29 (85)	24 (89)	0.97
Caregivers, parents	29 (85)	22 (82)	0.74
Family income (Baht per month)	40,000 ± 23,000	36,400 ± 25,000	0.23
Maternal education, bachelor, n (%)	30 (88)	19 (70)	0.11

**Table 4:** Multivariate analysis of factors associated with vitamin D insufficiency (< 20 ng/mL) in the study infants

Variable	Crude Odds Ratio (95%CI)	*p-value*	Adjusted Odds Ratio (95%CI)	*p-value*
Duration of sunlight exposure per day				
> 15 min	Reference		Reference	
≤ 15 min	1.83 (1.06–3.17)	< 0.01	1.37 (0.81–1.68)	0.41
Duration of sunlight exposure per weeks				
> 100 min	Reference		Reference	
≤ 100 min	12.15 (2.89–84.4)	< 0.01	5.61 (1.87–18.38)	< 0.01
Maternal vitamin D insufficiency				
> 20 ng/mL	Reference		Reference	
≤ 20 ng/mL	19.12 (3.93–160.75)	< 0.01	9.00 (2.54–43.09)	< 0.01

## Discussion

The overall prevalence of VDI in our study infants was high at 28.3%, all of whom were mainly breastfed. None of the mainly formula-fed infants in our study had VDI as their daily averagely milk intake was 888 mL/day of vitamin-D fortified formula, an amount which nearly provided the 400 IU of recommended daily vitamin D intake. Therefore, main breastfeeding infant whose mother had VDI was the main risk factor for VDI. Focusing on the mainly breastfed infants, the prevalence of VDI was very high at 56%. Another factor significantly associated with VDI in our study was the duration of sunlight exposure, as the longer the duration of sunlight exposure, the higher the serum 25OHD level. The time of day the parents preferred to bring their infants for sunlight exposure was in the early morning or in the late afternoon.

Over the last 20 years, high prevalence of infantile nutritional rickets have been reported in many countries, both developed and developing [8, 9, 13, 14]. Recent studies of vitamin D status in Southeast Asian countries, including Thailand, by South East Asian Nutrition Surveys (SEANUTS) in children aged 3–10 years using the classifications of VDD of < 20 ng/mL and VDI of 20–29 ng/mL have found prevalence of VDI (including VDD) as high as 50% [8, 9]. The factors associated with VDI reported in these surveys were exclusively being breastfed as infants, insufficient daytime outdoor activities, use of sunscreen and maternal VDI. To our knowledge, our work is the first report in Thailand, and maybe in Southeast Asia, studying the vitamin D levels in 6–12-month-old infants, along with other biochemical parameters related to vitamin D metabolism, and also the timing and duration of sunlight exposure in infants.

Although human breast milk is highly nutritious, containing adequate amounts of most important vitamins and immunological factors, it contains only low levels of certain micronutrients such as vitamin D, iron, and vitamin K [6]. Human breast milk contains only 15–50 IU/L of vitamin D, and even lower amounts in vitamin D-deficient nursing mothers [6, 7]. The amount of vitamin D transferred from mother to infant through human breast milk varies depending on the maternal dietary intake and serum 25OHD concentration [15]. Our study found a significant association between VDI breastfed infants and VDI nursing mothers, which was similar to studies in other Southeast Asian countries [8, 9]. The AAP in 2008, and more recently the Global Consensus 2016, recommend that exclusively breastfed infants should be given 400 IU of vitamin D daily beginning in the first week of life and this 400 IU vitamin D daily supplementation should be continued until the infant is receiving at least 1000 mL per day of 400 IU-vitamin D-fortified formula [12]. Several trial studies of supplementing 4,000–6,400 IU vitamin D3 daily for 6 months in nursing mothers reported that this was safe for mothers and provided higher maternal circulating 25OHD levels to meet infant requirements without adverse events [15–17]. However, the global consensus 2016 recommends that lactating women should take dietary vitamin D supplementation of 600 IU per day for their own needs rather than a high daily dose of 4,000–6,400 IU vitamin D for the needs of both the mothers and their infants [12]. At present in Thailand, there is no national policy or campaign for vitamin D supplementation in either exclusively breastfed infants or their nursing mothers.

The other main source of vitamin D is cutaneous vitamin D synthesis from UVB sunlight exposure [18, 19]. The time for the most efficient UVB radiation for vitamin D synthesis is 10.00–15.00 h with 15 min of sunlight exposure on one-fourth of the body area (arms and face, or arms/hands and legs), which can produce adequate vitamin D in light-skinned populations [18–21]. A review by the AAP in 2008 concluded that 2 h of sunlight exposure per week in fully clothed infants without a hat can maintain a sufficient vitamin D level of > 11 ng/mL in white infants [20]. Various studies have concluded that the Asian skin types require 2–3 times more solar UVB exposure than Caucasians [19, 21–23]. The cutaneous synthesis of vitamin D varies in populations depending on latitude of the study area, seasonal variations of sunshine hours, time of day and duration of sunlight exposure, skin color, skin coverage by clothing, and sunscreen use. At present, there are no recommendations or suggestions for the appropriate time of the day or the adequate duration for sunlight exposure in infancy and childhood age groups. In our study, not all mainly breastfed infants had VDI, indicating that a low vitamin D level from inadequate vitamin D intake could be partially compensated for by adequate duration of sunlight exposure for cutaneous vitamin D synthesis in either the early morning or late afternoon. Although the peak time for cutaneous vitamin D synthesis is between 10.00–15.00 h, this is also the time of the highest daytime temperature of 30–35 degrees Celsius in southern Thailand and most people avoid going outdoors during that period, as our study found that only 4% of the mothers or caregivers brought their infants outdoors during the 10.00–15.00 h for less than 5 min. Moreover, exposure to UV rays from sunlight during the middle period of the day can be hazardous for young children and infants [24]. We postulate that the adequate cutaneous vitamin D synthesis in our infants despite less UBV exposure in the early morning or late afternoon was because the skin of infants is not as thick nor as dark as adult skin and none of our infants had sunscreen applied. Also, most of the infants in our study were normally dressed only in short-sleeved shirts and short pants due to the normally warm weather in Thailand, and thus the percentage of skin exposed to sunlight was relatively greater compared to older children and adults exposure areas.

Our study had both some strengths and limitations. The main strength was that it is the first study in Thailand focusing on serum levels of vitamin D with other biochemical parameters related to vitamin D status in infants aged 6–12 months and their mothers along with the feeding type and the time and duration of sunlight exposure. Our study provides useful information on the duration of sunlight exposure in mainly breastfed infants which is probably an important factor in preventing VDI. The limitations were, first, this was a hospital-based study in a tertiary medical center in which selection bias could have occurred as most of the participants were educated and lived and worked in an urban area with relatively high family incomes, which does not represent the general Thai population. Second, we used a structured questionnaire to collect data on infant feeding and sunlight exposure which could be biased by under- or over-reporting of the parent’s self-reporting. We were aware of such potential errors, however, and we compensated for these potential biases by in-depth interviews particularly concerning the time of the day of going outdoors, duration sunlight exposure in minutes per day, and the days per week of sunlight exposure in each mother to ensure a high level of accuracy of the information. Third, the serum total 25OHD measurements were performed by radioimmunoassay using a Liaison®; DiaSorin-Radioimmunoassay method, which is not a reference method for 25OHD determination in research studies as compared to the high-performance liquid chromatography (HPLC) or liquid chromatography-tandem mass spectrometers LC–MS/MS. However, there have been many studies showing the accuracy with good agreement of the Liaison® radioimmunoassay method and HPLC or LC–MS/MS methods (*r*^*2*^ = 0.91-0.95) [25, 26]. Lastly, skin color of the infants in our study was not assessed, which could have had some effects on cutaneous vitamin D synthesis.

In summary, our findings suggest that the prevalence of VDI was high in mainly breastfed infants whose mothers had VDI in our study area in Southern Thailand, and that the infants with duration of sunlight exposure in the early morning or in the late afternoon of at least 100 min per week or 15 min per day have adequate 25OHD levels. Sunlight exposure duration and mainly breastfeeding from VDI mothers were significant factors related to VDI in healthy term infants. As recommended by the 2016 Global Consensus Recommendations on Prevention and Management of Nutritional Rickets, 400 IU of vitamin D should be given daily to exclusively breastfed infants to prevent vitamin D insufficiency and the nursing mother should take dietary vitamin D supplementation of 600 IU per day.

## Data Availability

The data are not publicly available due to it was funded by the Southern Thailand Institute of Research and Development and some data contained information that could compromise the privacy of research participants However, The data used to support the findings of this study are available from the corresponding author (SJ) upon request.

## References

[1] Chang SW, Lee HC (2019). Vitamin D and health - The missing vitamin in humans. Pediatr Neonatol.

[2] Stoffman N, Gordon CM (2009). Vitamin D and adolescents: what do we know?. Curr Opin Pediatr.

[3] Lessen R, Kavanagh K (2015). Position of the academy of nutrition and dietetics: promoting and supporting breastfeeding. J Acad Nutr Diet.

[4] World Health Organization (2002). Nutrient adequacy of exclusive breastfeeding for the term infant during the first six months of life.

[5] Thai Health Project. The 2017 Infant and Young Child Food Marketing Control Act. A preliminary victory for Thai mothers and children; Thai Health 2018. Nakorn Pathom: Institute for Population and Social Research, Mahidol University; 2018. p. 50–4. [Internet]. Available from http://www.hiso.or.th/hiso/picture/reportHealth/ThaiHealth2018/eng2018_16.pdf.

[6] Erick M (2018). Breast milk is conditionally perfect. Med Hypotheses.

[7] Keikha M, Bahreynian M, Saleki M, Kelishadi R (2017). Macro- and micronutrients of human milk composition: Are they related to maternal diet? A comprehensive systematic review. Breastfeed Med.

[8] Poh BK, Rojroongwasinkul N, Nguyen BK, Sandjaja, Ruzita AT, Yamborisut U, et al; SEANUTS Study Group. 25-hydroxy-vitamin D demography and the risk of vitamin D insufficiency in the South East Asian Nutrition Surveys (SEANUTS). Asia Pac J Clin Nutr. 2016;25:538–48. 10.6133/apjcn.092015.02 27440689

[9] Rojroongwasinkul N, Kijboonchoo K, Wimonpeerapattana W, Purttiponthanee S, Yamborisut U, Boonpraderm A (2013). SEANUTS: the nutritional status and dietary intakes of 0.5–12-year-old Thai children. Br J Nutr.

[10] Wikipedia. Hat Yai. Available from: https://en.wikipedia.org/wiki/Hat Yai. [Cited 14 2022 Apr].

[11] World Health Organization. WHO child growth standards. Available from: https://www.who.int/tools/child-growth-standards. [Cited 14 Apr 2022].

[12] Munns CF, Shaw N, Kiely M, Specker BL, Thacher TD, Ozono K (2016). Global consensus recommendations on prevention and management of nutritional rickets. J Clin Endocrinol Metab.

[13] van Schoor N, Lips P (2017). Global overview of vitamin D status. Endocrinol Metab Clin North Am.

[14] van der Pligt P, Willcox J, Szymlek-Gay EA, Murray E, Worsley A, Daly RM (2018). Associations of maternal vitamin D deficiency with pregnancy and neonatal complications in developing countries: A systematic review. Nutrients.

[15] Keikha M, Bahreynian M, Saleki M, Kelishadi R (2017). Macro- and micronutrients of human milk composition: Are they related to maternal diet?. A comprehensive systematic review Breastfeed Med.

[16] Hollis BW, Wagner CL, Howard CR, Ebeling M, Shary JR, Smith PG (2015). Maternal versus infant vitamin D supplementation during lactation: a randomized controlled trial. Pediatrics.

[17] Andrews L, Phlegar K, Baatz JE, Ebeling MD, Shary JR, Gregoski MJ (2022). Comparison of infant bone mineral content and density after infant daily oral vitamin D 400 IU supplementation versus nursing mother oral 6,400 IU supplementation: A randomized controlled lactation study. Breastfeed Med.

[18] Nair R, Maseeh A (2012). Vitamin D: The “sunshine” vitamin. J Pharmacol Pharmacother.

[19] Engelsen O (2010). The relationship between ultraviolet radiation exposure and vitamin D status. Nutrients.

[20] Misra M, Pacaud D, Petryk A, Collet-Solberg PF, Kappy M (2008). Drug and Therapeutics Committee of the Lawson Wilkins Pediatric Endocrine Society Vitamin D deficiency in children and its management: review of current knowledge and recommendations. Pediatrics.

[21] Bogh MKB (2012). Vitamin D production after UVB: Aspects of UV-radiated and personal factors. Scand J Clin Lab Invest.

[22] Nimitphong H, Holick MF (2013). Vitamin D status and sun exposure in Southeast Asia. Dermatoendocrinol.

[23] Jablonski NG, Chaplin G. human skin pigmentation as an adaptation to UV radiation. Proc Natl Acad Sci U S A. 2010;107 Suppl 2(Suppl2):8962–8. 10.1073/pnas.0914628107 PMC3024016 20445093

[24] American Academy of Pediatrics, Committee on Environmental Health. Ultraviolet light: a hazard to children. Pediatrics. 1999;104(2 Pt 1):328–33. 10429020

[25] Binkley N, Carter GD (2017). Toward clarity in clinical vitamin D status assessment: 25OHD assay standardization. Endocrino Metab Clin North Am.

[26] Farrell CJ, Herrmann M (2013). Determination of vitamin D and its metabolites. Best Prac Res Clin Endocrinol Metab.

